# Impact of cryopreservation on PBMC-derived CAR-T cell manufacturing outcomes: a systematic review

**DOI:** 10.3389/fimmu.2026.1876909

**Published:** 2026-07-21

**Authors:** Rama Amin Al-Wreidat, Sakhr Alshwayyat, Laith Ahmad Maharma, Maysa Al-Hussaini

**Affiliations:** 1 Department of Cell Therapy and Applied Genomics, King Hussein Cancer Center, Amman, Jordan; 2 Department of Surgery, King Hussein Cancer Center, Amman, Jordan

**Keywords:** CAR-T cell, cryopreservation, manufacturing, PBMC, systematic review

## Abstract

**Background:**

Chimeric antigen receptor T-cell (CAR-T) therapy is an established treatment for several hematological malignancies, with peripheral blood mononuclear cells (PBMCs) serving as the starting material for manufacturing. Cryopreservation of PBMCs may offer logistical flexibility, although its influence on manufacturing outcomes remains incompletely defined. This review aimed to compare the effect of fresh versus cryopreserved PBMC starting material on CAR-T cell manufacturing outcomes, including viability, fold expansion, and transduction efficiency.

**Methods:**

A systematic review was conducted following PRISMA guidelines. PubMed and Google Scholar were searched from inception through March 2026 for original studies comparing fresh and cryopreserved PBMCs in human CAR-T cell manufacturing. Methodological quality was assessed using the design-appropriate quality-appraisal tools, and findings were synthesized narratively due to heterogeneity in study design and protocols.

**Results:**

Five studies published between 2019 and 2025 met the inclusion criteria, comprising two clinical and three experimental analyses. Post-thaw viability and recovery of cryopreserved PBMCs ranged between 77% and 97%, slightly lower than fresh material. Fold expansion, transduction efficiency, and cytotoxic activity were generally comparable between groups, although some studies reported transient early differences including prolonged doubling times, mitochondrial dysfunction signals, and increased TIM-3 expression in cryopreserved-derived products.

**Conclusion:**

Cryopreservation can be considered a feasible approach in CAR-T manufacturing, with generally comparable outcomes despite early post-thaw cellular changes. These differences do not seem to consistently compromise the overall manufacturing performance. However, the current evidence remains limited and heterogeneous, and further studies are required to increase confidence in our initial findings.

## Graphical Abstract

**Figure:**
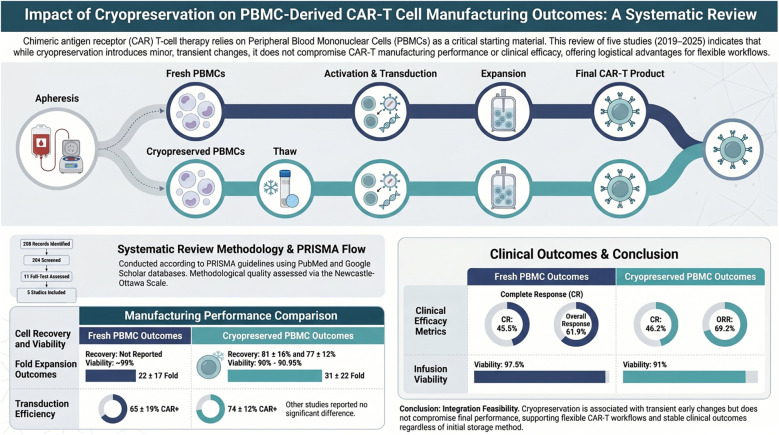

## 1 Introduction

Between 2017 and 2022, Chimeric Antigen Receptor T-cell (CAR-T) products received successive approvals by the United States Food and Drug Administration (FDA) for the treatment of several hematological malignancies including leukemia, lymphoma and multiple myeloma (1). Previous studies demonstrated this therapy as an effective treatment for follicular lymphoma and relapsed or refractory multiple myeloma with an overall response rate of 93% and 86% respectively (2, 3).

CAR-T cells are genetically engineered cells expressing a synthetic chimeric antigen receptor which recognizes tumor specific antigens independently of MHC molecule presentation. Upon antigen recognition, CAR-T cells become activated resulting in the initiation of strong immune responses against tumor cells (4). T-cell collection from peripheral blood is the first step involved in CAR-T cell production and is typically achieved through apheresis (5). After the collection process, T cells are activated and become prepared for CAR transgene delivery, which could be done via viral or non-viral methods. The genetically modified T cells are expanded *in vitro* to produce enough number of cells, then the final product is formulated and undergoes quality control and release testing prior to infusion (6).

The quality of the collected apheresis product could impact the effectiveness and success of CAR-T manufacturing, which is still a complex and highly variable procedure (7). For manufacturing and logistical reasons, apheresis-derived starting material may be cryopreserved prior to CAR-T cell production, providing additional time for product release and increasing access to more patients in different regions, while facilitating flexibility in processing and scheduling for shipping (8).

In spite of its potential benefits, cryopreservation of PBMC starting material may affect cell quality, as it can alter the cellular composition of the final CAR-T product, including increasing the percentage of regulatory T cells, which may ultimately impair the functionality and proliferative capacity of CAR-T cells *in vivo* (9). Moreover, freezing and thawing processes, along with variability in cryopreservation procedures, may negatively affect cell viability and recovery, thereby affecting downstream manufacturing outcomes (10).

The impact of using fresh versus cryopreserved PBMC as starting material on CAR-T cell production has not been clearly reported across previous studies. Therefore, this review aims to compare and study the effect of using fresh versus cryopreserved PBMC starting material in CAR-T cell production on manufacturing outcomes, including cell viability, transduction efficiency and fold expansion.

## 2 Materials and methods

### 2.1 Study design and search strategy

This systematic review assessed the impact of fresh versus cryopreserved peripheral blood mononuclear cells (PBMCs) as starting material for CAR-T cell manufacturing, in accordance with the PRISMA 2020 guidelines.

PubMed and Google Scholar were searched from inception until March 2026. Keywords included “Chimeric Antigen Receptor T-Cell”, “CAR-T”, “Peripheral Blood Mononuclear Cells”, “PBMC”, “cryopreservation”, “cryopreserved”, “fresh”, “manufacturing”, “viability”, “expansion”, and “yield”, combined using Boolean operators (AND, OR). Only studies published in English were included. The final search was completed on 01 March 2026. All retrieved records were imported into Rayyan software for screening. Then duplicate records were removed before starting the two-stage screening (title/abstract screening followed by full-text screening) based on predefined criteria. Reference lists of the included studies were screened; however, no additional eligible studies were identified. The complete search strategies are provided in **Supplementary Table 1**. The review protocol was not prospectively registered.

#### 2.1.1 Review question (PICO framework)

The review question was developed according to PICO framework. The population included all studies that involved PBMCs used as starting material for CAR-T cell manufacturing. The intervention was the use of cryopreserved PBMCs as starting material for CAR-T cell manufacturing. Studies that used only cryopreserved PBMCs without any comparator group were excluded. The comparator was fresh, non-cryopreserved PBMC starting material. The primary outcome of this study was cell viability, while secondary outcomes included fold expansion and transduction efficiency.

### 2.2 Eligibility criteria and outcomes

Studies were included if they were original research articles comparing fresh and cryopreserved PBMCs for human CAR-T cell manufacturing and reported manufacturing-related outcomes. The primary outcome was cell viability; secondary outcomes were fold expansion and transduction efficiency.

Studies were excluded if they were reviews, lacked a direct comparison between fresh and cryopreserved PBMCs, used animal models, or did not report CAR-T manufacturing outcomes.

All identified records were imported into Rayyan software for screening. After removing duplicates, titles and abstracts were screened independently. Data extraction was performed independently by SA and RA using a standardized form. Disagreements were resolved through discussion or consultation with a third reviewer. Extracted data included first author, year of publication, study design, target antigen, source of PBMCs, type of starting material (fresh versus cryopreserved), manufacturing characteristics, and outcomes including cell viability, fold expansion, and transduction efficiency.

For studies reporting multiple manufacturing stages, only data related to cryopreservation of PBMC or leukapheresis starting material were included in the primary analysis and data related to cryopreservation of final CAR-T cell products were reported descriptively where available but not included in comparative synthesis.

### 2.3 Quality assessment and data synthesis

Because the included studies differed in design, methodological quality was appraised using instruments suited to each study type. The two clinical comparative studies were assessed using the Newcastle–Ottawa Scale (NOS) (11), which evaluates study quality across three domains: selection of study groups, comparability between groups, and outcome assessment, with a maximum score of nine points and studies scoring seven or more considered high quality. The three experimental manufacturing studies were appraised using a structured quality checklist adapted for *in vitro* work, covering clarity of study objectives, characterization of donor and sample material, adequacy of methodological reporting, objectivity of outcome measurement, and use of appropriate replicates or controls. Each experimental study was rated as low, moderate, or high methodological concern. The results of both appraisals are summarized in **Table 1**.

**Table 1:** Methodological quality appraisal of the included studies. Clinical comparative studies were appraised with the Newcastle–Ottawa Scale (panel A); experimental manufacturing studies were appraised with a structured checklist adapted for *in vitro* studies (panel B).

(A) Clinical comparative studies (Newcastle–Ottawa Scale).						
Study	Selection (max 4)	Comparability (max 2)	Outcome (max 3)	Total (/9)	Overall quality	
Brezinger-Dayan et al. (13)	3	2	3	8	High
Panch et al. (12)	4	2	2	8	High
(B) Experimental manufacturing studies (structured *in vitro* checklist; each domain rated as low, moderate, or high concern).						
Study	Objectives	Donor/sample characterization	Methodological reporting	Outcome measurement	Replicates/controls	Overall concern
Xu et al. (15)	Low	Moderate	High	Low	Moderate	Moderate
Ren et al. (20)	Low	Low	Low	Low	Low	Low
Abraham-Miranda et al. (14)	Low	Low	Low	Low	Moderate	Low

Due to heterogeneity in study designs, manufacturing protocols and reported results, meta-analysis could not be conducted. Findings were consolidated narratively, focusing on differences between fresh and cryopreserved PBMCs in cell viability, fold expansion, and transduction efficiency.

## 3 Results

### 3.1 Study selection and characteristics of included studies

**Figure 1:**
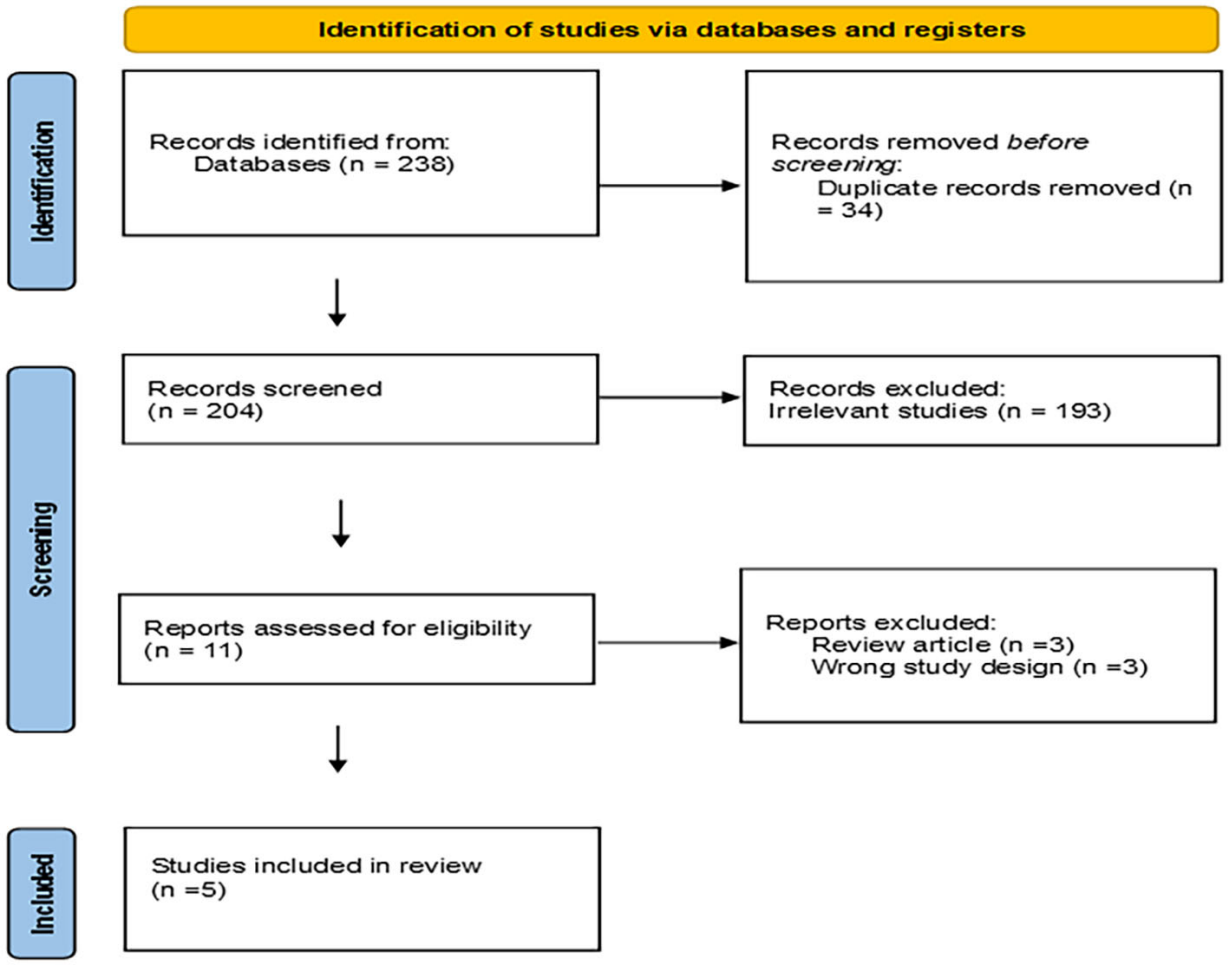
Selection of studies from the systematic search of the literature.

Literature search identified 238 records from PubMed and Google Scholar. After removing duplicates, the remaining 204 studies underwent title and abstract screening. Of these, 11 studies were assessed for full-text eligibility. Only five studies met the inclusion criteria and were included in the final analysis (**Figure 1**).

The included studies, which were published between 2019 and 2025, comprise two clinical manufacturing analyses and three experimental laboratory investigations. Most of them focused on CD19-targeted CAR-T cells; one evaluated mesothelin-targeted cells, and another assessed multiple CAR platforms. Among the included studies, the primary analysis focused on fresh versus cryopreserved PBMCs as starting material. One study evaluated cryopreservation of leukapheresis products, and two studies additionally reported outcomes for cryopreserved final CAR-T cell products; these were considered separately for descriptive purposes. Study characteristics are summarized in **Table 2**.

**Table 2:** Characteristics of included studies and reported outcomes comparing fresh and cryopreserved starting materials for CAR-T cell manufacturing.

Panel A — Study characteristics.							
Study	Design	Starting material	Cryo stage	CAR target	Gene transfer	Culture platform	
Brezinger-Dayan (13)	Clinical	PBMC	PBMC (+ final product, descriptive)	CD19	Gamma-retroviral vector	G-Rex100/T175
Panch (12)	Clinical	PBMC	PBMC (+ final product, descriptive)	Multiple platforms	Viral transduction	Ex vivo expansion in standard culture bags/flasks
Xu (15)	Experimental	PBMC	PBMC	CD19	PiggyBac (transposon)	AIM-V static culture
Ren (20)	Experimental	Leukapheresis	Leukapheresis	Multiple platforms*	Viral (lentiviral/retroviral), Non-viral	G-Rex
Abraham-Miranda (14)	Experimental	PBMC	PBMC	CD19	Lentiviral transduction	Anti-CD3/CD28 activated ex vivo expansion
Panel B — Outcomes.							
Study	Viability	Recovery	Expansion	Transduction	Phenotype	Cytotoxicity	Clinical outcome
Brezinger-Dayan	NR	81 ± 16% (PBMC)	Day-10: 22 ± 17 (fresh) vs 31 ± 22 (cryo)	65 ± 19% vs 74 ± 12%	↑ TIM-3	NR	No sig. difference in response rates
Panch	NR	~77 ± 12%	Comparable; lower early counts	No sig. effect	Mitochondrial dysfunction/apoptosis signals	NR	Comparable persistence & outcomes
Xu	~90.95%	NR	~21-fold	Consistent	NR	Comparable tumor-killing	CR 46.2% vs 45.5%; ORR 69.2% vs 61.9%
Ren	>90% (fresh ~99%)	T-cell 97.5% vs 70.0%	NR	No sig. differences	↑ lymphocyte proportion (66.59% vs 52.20%)	NR	No clinical data
Abraham-Miranda	NR	NR	↑ doubling time; comparable final	NR	↓ IFN-γ	Comparable vs CD19; similar cytokines	None (*in vitro*)

↑ indicates increased expression or level; ↓ indicates decreased expression or level.

### 3.2 Cell viability and recovery

Brezinger-Dayan et al. reported post-thaw recovery of 81 ± 16% for cryopreserved PBMCs and similar results were found by Panch et al., with post-thaw recovery of approximately 77 ± 12% for cryopreserved PBMNCs.

Among experimental studies, Xu et al. observed average PBMC viability of approximately 90.95% after cryopreservation, even after extended storage and Ren et al. reported post-thaw viability exceeding 90% for cryopreserved leukapheresis samples, slightly lower than fresh samples (approximately 99%). Overall, cryopreservation resulted in slight viability reductions but maintained sufficient recovery for successful CAR-T manufacturing. Where fresh PBMC viability was directly reported, it was high; Ren et al. recorded approximately 99% in fresh samples, providing a baseline against which the post-thaw values of approximately 77% to 97% can be interpreted. The recovery values reported by Brezinger-Dayan et al. and Panch et al. were expressed relative to this fresh baseline.

Outcomes related to final CAR-T cell products were reported in some studies. Brezinger-Dayan et al. described a post-thaw recovery of 77 ± 18% for cryopreserved CAR-T products after long-term storage, while Panch et al. reported a recovery of 97 ± 17% for CAR-T products. These findings are presented for descriptive context only and were not included in the comparative analysis of starting material cryopreservation.

In the included clinical studies, the final CAR-T cell products were not uniformly infused fresh; Brezinger-Dayan et al. and Panch et al. additionally reported cryopreserved final CAR-T products that were infused after thawing. This distinction is relevant because cryopreservation of the final product, which is separate from the PBMC starting material that is the focus of this review, may also influence product quality.

### 3.3 CAR-T cell expansion

Brezinger-Dayan et al. reported similar day-10 fold expansion in fresh and cryopreserved PBMC-derived CAR-T products (22 ± 17 vs 31 ± 22). Panch et al. found that cryopreservation did not significantly affect final expansion, although cultures from cryopreserved PBMNCs showed lower viable cell counts early in culture.

Xu et al. reported approximately 21-fold expansion from cryopreserved PBMCs, comparable to fresh cultures. Abraham-Miranda et al. observed modestly increased doubling time with cryopreserved PBMCs, indicating slower early proliferation, though final expansion remained comparable. Collectively, cryopreservation may affect early proliferation kinetics, but final CAR-T expansion is generally preserved.

### 3.4 Transduction efficiency and CAR expression

Brezinger-Dayan et al. reported CAR-positive proportions of approximately 65 ± 19% for fresh and 74 ± 12% for cryopreserved PBMC-derived products, with no significant difference. Panch et al. also found no significant effect of cryopreservation on transduction efficiency.

Xu et al. reported consistent CAR expression in fresh and cryopreserved groups using the PiggyBac system, and Ren et al. observed no significant differences across multiple manufacturing platforms. These findings indicate that cryopreservation preserves CAR gene transfer and expression levels.

### 3.5 Functional activity of CAR-T cells

Abraham-Miranda et al. found that CAR-T cells from cryopreserved PBMCs showed comparable cytotoxic activity against CD19-expressing tumor cells and similar cytokine production profiles. Xu et al. also reported comparable tumor-killing activity between groups.

Clinically, Brezinger-Dayan et al. did not find significant differences in response rates between patients receiving CAR-T cells from fresh versus cryopreserved materials. Panch et al. reported comparable *in vivo* persistence and clinical outcomes. In these few studies, cryopreservation was not associated with a clear reduction in cytotoxic function or clinical efficacy, although the clinical comparisons were limited and should be interpreted with caution.

## 4 Discussion

This review suggests that cryopreservation can be associated with measurable early cellular changes, especially in the kinetics of survival and proliferation, without clear evidence of significant impairment of final manufacturing performance. Among the studies included, fold expansion, transduction efficiency and functional activity were generally comparable between fresh and frozen groups despite some early post-thaw differences. These findings show that early isolated changes should be interpreted along with later manufacturing results instead of isolation.

Several of the included studies reported subclinical phenotypic and molecular changes after cryopreservation. Panch et al. (2019) identified elevated genetic characteristics associated with mitochondrial dysfunction and apoptosis signals in products from cryopreserved PBMCs, despite overall comparable downstream performance (12). Brezinger-Dayan et al. (2022) found that new CAR-T cells expressed significantly more TIM-3 and showed more *in vitro* tumor reactivity, although no major downstream differences were observed (13).

Abraham Miranda et al. (2022) reported an increased doubling time and a reduction in the secretion of IFN- in CD28-induced CAR-T cells generated from cryopreservation PBMCs, while cytolytic capacity remained unchanged (14). These findings suggest that cryopreservation can affect certain early cell characteristics, but these effects do not consistently compromise the broader manufacturing results assessed in available studies. The slightly higher CAR-positive proportion reported by Brezinger-Dayan et al. in cryopreserved compared with fresh products (74% versus 65%) did not reach statistical significance. It most likely reflects inter-sample variability and the small sample size rather than a true advantage of cryopreservation. A plausible contributing factor is that freeze-thaw stress may transiently favor more activated or proliferative T-cell subsets that are more readily transduced, although this interpretation remains speculative and was not directly tested in the original study.

Although clinical outcomes were not the primary focus of this review, the available reports didn’t reveal major differences between fresh and cryopreserved groups. For example, Xu et al. (2023) reported similar CR (46.2% vs 45.5%) and ORR (69.2% vs 61.9%), while Dreyzin et al. (2023) observed comparable response and toxicity profiles despite lower viability at infusion in the cryopreserved CAR-T product group (91% vs 97.5%) (15, 16). These clinical observations should be interpreted with caution. The available comparisons were limited in size and potentially confounded by differences in disease type, patient fitness, prior therapy, lymphodepletion, infused dose, CAR construct, and product phenotype, so they do not establish clinical equivalence between fresh and cryopreserved starting material.

From a practical point of view, cryopreservation allows cell collection, manufacturing and infusion to be coordinated more flexibly, which is relevant for centralized and decentralized CAR-T delivery models. In centralized systems, the vein-to-vein period is 22 days on average, and the manufacturing period could extend further (17). These delays can be clinically important for patients with aggressive diseases.

Decentralized point of care models have been proposed to reduce turnaround times and reduce production costs, and cryopreservation can facilitate such workflow adaptations by allowing flexible timescales for cell collection and processing. Cryopreservation also allows PBMC to be collected before introducing additional therapies, which can help preserve healthier T cells for future manufacture (14).

A previous study described a GMP-compliant cryopreservation protocol with a post-defrost efficiency of 98.8% and a recovery rate of 72.8% (18). In this context, it is believed that cryopreservation can support more flexible manufacturing models and may be particularly relevant for future efforts to expand CAR-T access to regions with limited central infrastructure.

Literature studies varied in design, starting materials, gene transfer methods, and cryopreservation protocols, which limits the direct comparison of studies. Two studies comprised clinical analyses using patient samples, while three used healthy donor cells, which may overestimate the production performance compared to patients heavily pretreated (19).

Ren et al. reported that cryopreservation products produce a higher proportion of lymphocytes (66.59% vs. 52.20%) and a better recovery of T cells (97.5% vs. 70.0%) than cryopreservation PBMCs, suggesting that the source material itself can influence the quality after decomposition (20).

The available evidence did not yield consistent results. A previous study reported a lower survival rate of cryopreserved anti-CD19/CD20 CAR-T products (63% compared to 93%, p 0.01) and a higher CR rate in fresh products (79% compared to 40%), but the clinical comparison was limited by the small sample size (17). This contradicts with other reports that show no measurable impact, suggesting that current evidence is not fully consistent across the settings and study designs. Ren et al. (2025) provided further assurances by validating the products of cryopreserved leukapheresis across several manufacturing platforms, although no direct clinical results data were available (20).

Across the studies examined, a consistent finding was that cryopreserved cells often show a transient early delay in proliferation, yet they ultimately achieve comparable expansion. Abraham-Miranda et al. (2022) observed longer doubling times, while Panch et al. (2019) reported delayed onset of cell death in the first few days of culture, indicating that some cells remain susceptible after thawing even though immediate post-thaw viability appears acceptable (12, 14). This pattern may explain why early differences are observed even when later production outcomes are broadly similar. It aligns with the idea of a temporary recovery period following freezing before proliferation normalizes. Jeurink et al. demonstrated that cryopreserved PBMCs reach the same proliferation stage as fresh PBMCs (21), and Sadeghi et al. showed a significant immediate drop in ATP after thawing, followed by substantial metabolic recovery within 48 hours (22).

These findings could be explained by the biological effects of cryopreservation exerted on PBMCs. During freezing, cryoprotectants such as dimethyl sulfoxide are used in order to decrease intracellular ice crystal formation and increase cell viability (23). However, the freezing-thawing procedure is known to cause osmotic stress and transient damage to the cell membranes, which leads to low viability and apoptosis of the cells right after thawing (24). In addition, cryopreservation may temporarily alter cellular metabolism and immune-cell function before recovery in culture (25). These mechanisms may explain the transient post-thaw differences observed in some studies, particularly in cell viability and early proliferative capacity without consistently affecting downstream CAR-T manufacturing outcomes.

The review has notable strengths as it tackles a clinically relevant issue in CAR-T manufacturing and combines clinical and experimental data. Nonetheless, the evidence base is limited and heterogeneous, and differences in methodology and populations may restrict comparability and generalizability. Consequently, the findings should be interpreted cautiously until confirmed by larger, standardized studies.

## 5 Conclusion

Cryopreservation can be considered a feasible approach for CAR-T cell manufacturing and is generally associated with comparable outcomes despite some early post-thaw cellular changes. These early differences do not seem to consistently compromise the overall manufacturing performance, although the evidence remains limited and heterogeneous. Future studies should use larger, standardized, and directly comparable designs which assess both manufacturing and clinical outcomes to better define the role of cryopreservation in routine CAR-T production.

## Supplementary material

Supplementary Table 1 Search Strategy.
