# Supplementary material for: Impact of cryopreservation on PBMC-derived CAR-T cell manufacturing outcomes: a systematic review
Source: Front Immunol. 2026 Jul 21;17:1876909. doi: 10.3389/fimmu.2026.1876909 (PMC13433609; doi:10.3389/fimmu.2026.1876909)
Supplement: Supplementary file 1 [file Table1.docx]

**Appendix A**

Supplementary Table 1: Search Strategy

| **Database** | **Search strategy** |
| --- | --- |
| **PubMed** | (Chimeric Antigen Receptor T-Cell Therapy[MeSH] OR “CAR T cell”[tiab] OR “CAR-T”[tiab]) AND (Peripheral Blood Mononuclear Cells[MeSH] OR PBMC[tiab]) AND (Cryopreservation[MeSH] OR cryopreserved[tiab] OR fresh[tiab]) AND (manufacturing[tiab] OR “starting material”[tiab] OR viability[tiab] OR expansion[tiab] OR yield[tiab]) |
| **Google Scholar** | “CAR-T” AND PBMC AND (cryopreservation OR cryopreserved OR fresh) AND manufacturing |
